# Variability in L2 Vowel Production: Different Elicitation Methods Affect Individual Speakers Differently

**DOI:** 10.3389/fpsyg.2022.916736

**Published:** 2022-07-13

**Authors:** Murray J. Munro

**Affiliations:** Department of Linguistics, Simon Fraser University, Burnaby, BC, Canada

**Keywords:** Cantonese, second language acquisition, phonetics, ESL, intelligibility, vowels

## Abstract

Elicitation methods are known to influence second language speech production. For teachers and language assessors, awareness of such effects is essential to accurate interpretations of testing outcomes. For speech researchers, understanding why one method gives better performance than another may yield insights into how second-language phonological knowledge is acquired, stored, and retrieved. Given these concerns, this investigation compared L2 vowel intelligibility on two elicitation tasks and determined the degree to which differences generalized across vowels, vowels in context, lexical items, and individual speakers. The dependent variable was the intelligibility of Cantonese speakers' productions of English /i I u ℧/ in varying phonetic environments. In a picture-naming task, the speakers produced responses without an auditory prompt. In a second task–interrupted repetition–they heard exemplars of the same targets without pictures, and repeated each one after counting aloud to 10, a step intended to disrupt their short-term auditory store and therefore prevent simple mimicry. For target words with scores below 80% on picture naming, mean intelligibility was more than 10 points higher on interrupted repetition. However, that difference did not generalize across conditions or across speakers. Thus, although it is technically accurate to say that, on average, interrupted repetition yielded better vowel intelligibility than did picture naming, that observation requires a great deal of qualification, particularly because of individual speaker differences. The outcomes are interpreted in terms of their relevance to language assessment and phonetic learning.

## Introduction

The research presented here is part of a larger investigation of factors influencing vowel production by second-language (L2) speakers of English. The project was not originally motivated by any specific theoretical orientation on L2 production, but instead by pedagogical considerations. However, as will be shown, its relevance extends beyond that domain. Its central concern was the degree of uniformity in L2 vowel acquisition among learners sharing an L1 background. On the one hand, if very similar difficulties are experienced by many learners in a classroom setting, the workload of the pronunciation instructor is considerably lightened. Problem areas ought to be predictable in advance, and difficulties for most or all class members should be addressable with a common set of instructional activities, perhaps carried out in a lock-step fashion. On the other, a lack of uniformity suggests that a “one size fits all” approach to pronunciation teaching is inadequate and that individual differences require detailed attention if instruction is to be effective. Logically, of course, it might turn out that some aspects of pronunciation learning do show relative uniformity (at least for speakers with a shared L1 background), but that others do not. The project therefore is not aimed at making broad generalizations about L2 segments and prosody, but instead focuses on one specific area of concern: English high vowel acquisition by Cantonese speakers. That focus is appropriate because differences in the two languages' vowel inventories appear to underpin known difficulties for Cantonese speakers (Meng et al., 2007; Wong, 2015). Also, as observed by Cebrian et al. (2021), the English high vowel contrast between /i/ and /I/ has been the subject of much interest in L2 phonetics research, first because it has a high functional load in English, distinguishing many pairs of common words such as *heat* and *hit* (Levis and Cortes, 2008; Sewell, 2017). Second, it poses perceptual and productive difficulties for speakers from diverse L1 backgrounds, including Catalan, Mandarin, Russian, and Spanish (Mora and Fullana, 2007; Kondaurova and Francis, 2008; Munro and Derwing, 2008).

In addition to its pedagogical relevance, this examination of elicitation effects also offers theoretical promise in that it may provide insights into L2 acquisition mechanisms. In particular, evidence that one mode of elicitation yields better performance than the other would raise interesting questions about the processes involved in storing, retrieving, and implementing L2 phonological knowledge. Given the earlier set of findings, one relevant issue is whether individual L2 speakers show differential task effects. A comprehensive model of L2 phonetic learning would need to account for such variability.

The degree to which Cantonese and other learners of English diverge from one another in their success in high vowel production had received little attention until recently. Using speech elicited in a picture-naming task, Munro (2021) observed considerable interspeaker variability in the vowel intelligibility of Cantonese speakers when productions were considered in terms of vowels alone, vowels in rhymes, and even vowels within particular words. In this follow-up study, the Munro (2021) investigation is extended to compare the effect of two speech elicitation techniques, one with and one without an audio prompt, on interspeaker variability. The method of elicitation is important because accurately assessing learner difficulties is fundamental to both pedagogy and theory-building.

### Factors Influencing L2 Segmental Production

Pronunciation specialists have devoted considerable attention to the wide range of factors that might predict in advance or explain in *post-hoc* fashion L2 learners' difficulties in producing particular consonants or vowels. Mid-twentieth-century authorities attempted to justify such work by claiming that predicting phonological difficulties can improve pedagogical practices (Moulton, 1962). Although that opinion was disputed long ago (Walz, 1980), interest among teachers in error prediction has persisted (Munro, 2018; Rehman et al., 2020). Recently, Munro et al. (2015) and Munro (2018, 2021) discussed evidence that individual variability in L2 production, even among speakers of a shared L1, is greater than has sometimes been assumed. Although it may be possible to offer broad, probabilistic error hierarchies for groups of learners from particular backgrounds, such predictions often do not apply to all, or even to the majority, of learners.

Influences on L2 pronunciation may be classified as linguistic or non-linguistic. By “linguistic,” I mean those that relate specifically to one or both languages at issue. Chief among these is the degree of correspondence between the phonological systems of the languages (Lado, 1957), which is said to trigger “negative transfer” when structures differ. Although transfer effects are clearly an important influence on L2 segmental accuracy, a purely transfer-based account of errors is unsatisfactory, as discovered in early investigations of the Contrastive Analysis Hypothesis (Brière, 1966; Wardaugh, 1970). To some degree, this inadequacy may be due to faulty approaches to comparing language inventories (see Flege and Bohn, 2021; Thomson, 2021). Furthermore, different speakers may differ in their phonetic representations of the sound segments of L1, and may therefore relate L2 sounds to L1 sounds in idiosyncratic ways. Also, the assumption that “what is different is difficult” is undermined by research indicating that similarity, rather than difference, can pose serious problems in L2 phonetic learning (Flege, 1987).

From a theoretical standpoint, invoking “transfer” entails a lack of specificity about the underlying cognitive mechanisms involved in acquiring, storing, and activating phonetic knowledge. Some theorists have attempted to refine the transfer concept to incorporate other linguistic factors predictive of learning (see Archibald, 2021, for a review). A variety of proposals have been offered that integrate such concepts as markedness (Eckman, 1985; Major and Kim, 1996), language-specific constraint rankings (Lombardi, 2003), and feature geometry (Brown, 2000). The extent to which such approaches improve predictive success is not at issue here. Rather, the starting point for the current study is the evidence of considerable inter-speaker variability in phonetic acquisition. This is not generally a focus of linguistic modeling *per se*, and for the most part, such variability must be the result of something other than linguistic factors.

Non-linguistic factors are often highlighted in research on individual differences in L2 phonetic learning. These are independent of the specific languages at issue (for reviews see Piske et al., 2001; Mora, 2022). For instance, the age of L2 learning (AOL) correlates negatively with foreign accent ratings (Flege et al., 1995, 1999; Bylund et al., 2021). Aptitude and motivational factors are also implicated in phonetic learning success (Perrachione et al., 2011; Hu et al., 2013; Kissling, 2014; Nagle, 2018b), as has the quantity of L2 experience (see Flege and Bohn, 2021; Flege et al., 2021). These factors affect global aspects of L2 pronunciation such as accentedness and intelligibility and figure prominently in some theoretical approaches. In particular, the Speech Learning Model (SLM, Flege, 1995) and the Revised Speech Learning Model (SLM-r, Flege and Bohn, 2021) emphasize language experience effects. However, such influences are of much less importance (or are not useful at all) in predicting specific phonetic problems, such as vowel or consonant errors. Munro et al. (1996), for example, observed a negative relationship between AOL and L2 vowel goodness in Italian speakers of English, with variable effects from vowel to vowel for both accuracy and intelligibility. Moreover, different speakers with approximately the same AOL, and similar L2 experience, varied in the number of the 11 target vowels they produced intelligibly, with some producing as few as six and others producing all 11 intelligibly. This led Munro et al. (1996, p. 332) to observe that “between-vowel effects did not occur uniformly for all, or even for a large majority, of the learners.” Such variability, as well as the parallel lack of uniformity in Munro (2021), does not appear explicable in terms of aptitude, motivation, or quantity of general L2 experience. In particular, there is no obvious reason why such characteristics should lead one speaker to produce good exemplars of /I/ in *hit*, but mostly unintelligible productions of the same vowel in *sit*, while another speaker shows the opposite pattern. Yet, just such disparities were seen in the study by Munro (2021), suggesting that learners' knowledge of particular lexical items plays a role in vowel production accuracy. In an investigation of Korean speakers' English productions, Baker and Trofimovich (2008) found an advantage for vowels in words of higher frequency and greater subjective familiarity among adult speakers. Acquired vowel knowledge may therefore depend on the quality, quantity, and timing of learners' encounters with particular words. Hypothetically, for instance, frequent experience with a word very early in the L2 acquisition process, when control over the pronunciation of L2 structures is limited, might yield a different learning outcome from exposure at a later time. Given that L2 phonological knowledge develops rapidly during the hypothesized Window of Maximal Opportunity at the first massive exposure to L2 (Derwing and Munro, 2015), a timing difference of weeks or even days may affect word learning in important ways.

### Task Effects in L2 Production

Teachers, assessors, and researchers elicit L2 speech in a variety of ways, depending on their goals. When approximation of real-world language is paramount, extemporaneous and interactive production tasks are preferred, though a drawback of these is limited researcher control over phonetic content. In the assessment of segmental production–a focus of the present study–the elicitation task must yield enough usable exemplars of the target sound to allow satisfactory analysis. Possible tasks include simple repetition (Flege and Eefting, 1988), reading aloud, delayed repetition, in which the speaker reformulates an utterance with a target item (Flege et al., 1995, 1999; Munro and Derwing, 2008), picture naming (Flege and Davidian, 1984; Cebrian et al., 2021), or less-constrained tasks, such as timed picture descriptions (TPD), in which speakers are instructed to use particular target items while giving their descriptions (Saito and Munro, 2014).

Ample evidence shows that differences in elicitation methods can affect L2 production. In comparison with simple repetition, read-aloud tasks can lead to stronger orthographic influences on pronunciation (Bassetti and Atkinson, 2015), even in familiar words produced by experienced L2 users. It is worth noting as well that non-reading tasks do not necessarily eliminate orthographic effects because speakers may have developed internalized representations of words by assuming and practicing pronunciations based on spelling. In fact, even native speakers sometimes use “reading pronunciations” of orthographically opaque words like *epitome* and *blackguard*.

In immediate word repetition, the availability of an aural model appears to facilitate accurate production. Rojczyk (2013), for example, found that Polish speakers produced more native-like English /æ/ formants in immediate imitation than in list reading. Although that seems to suggest that repetition does not require speakers to access their own phonological category representations, Llompart and Reinisch (2019) have argued against such a view. In fact, they observed a close link between speakers' imitation performance and their perceptual capabilities. They also found, however, that imitated and read words containing a difficult vowel distinction for German learners of English differed noticeably in their acoustic properties. They attributed the lower accuracy in reading to “inaccurate non-native lexical representations” (p. 594), which were accessed during reading but not in repetition.

Saito and Munro (2014) performed acoustic analyses on word-initial English /ɹ/ produced by groups of Japanese speakers either living in Japan or residing in Canada for 1–12 months. Targets were elicited in word-reading, sentence-reading, and TPD conditions. In apparent contrast with the findings discussed above, native-like F3 values were found in word-reading, though this was true only for speaker groups with 5 or more months of residency, and the other acoustic dimensions (F2, F1 transition duration) remained unaffected by the task. The authors proposed that the difference was due to the speakers' use of a controlled mode of production during reading.

Also of potential relevance to task effects is research on auditory priming. Trofimovich and Gatbonton (2006) exposed listeners to spoken target words in a priming block followed by a distractor task and a subsequent speeded repetition task. The previously heard targets were produced more quickly than items not heard during the priming block. That outcome suggests that activation during priming persisted even after the distractor task and facilitated access to relevant lexical representations for production. Following on that study, Leong et al. (2021) investigated Mandarin speakers' productions of English tense and lax high vowels, noting that Mandarin makes no tense-lax distinction. Target words were elicited *via* an orthographic presentation on a screen and were primed with a recorded vowel production (three iterations) that either matched (congruent condition) or did not match (incongruent condition) the vowel in the CVC target. A listener-based assessment revealed that /i/ and /I/ were produced with higher intelligibility in the congruent than in the incongruent condition, though a parallel finding was not obtained for the high back vowels. Because the same speakers showed some ability to distinguish tense and lax vowels perceptually, Leong et al. (2021) appealed to a perception-based explanation. In this case, priming with congruent vowels may have facilitated access to the correct perceptual representations for the targets. In fact, most theorizing about L2 speech learning assumes a relationship between perception and production, particularly given evidence that perceptual learning can lead to more accurate production (Nagle, 2018a). Notably, however, the SLM-r (Flege and Bohn, 2021) has abandoned the original SLM's assumption that accurate perception is a precondition for accurate production, in favor of the view that the two co-evolve through bi-directional processes.

### The Present Study

This study extends Munro (2021) with a parallel design involving the same participants, with the addition of a new variable: the elicitation method. The focus is the intelligibility of English high vowel productions of Cantonese speakers under two conditions: a picture-naming task (reported in Munro, 2021) and an interrupted repetition task.

#### Comparison of Cantonese and English Vowels

As a result of Hong Kong's historical status as a British Overseas Territory, English is one of its official languages, and a Hong Kong variety of English has emerged (Hung, 2000; Hansen Edwards, 2015; Sewell, 2022). The vast majority of Hong Kong residents speak Cantonese natively, with many having a native command of Hong Kong English (HKE) as well. At the same time, some residents are speakers of English as a second language, having grown up with little experience using English for social purposes or work-related communication. Speakers from the latter demographic who had immigrated to Canada were targeted for this investigation.

Whereas Western Canadian English (WCE) has four contrastive high vowels differing in advancement and “tenseness” (/i I u ℧/), Cantonese has /i/ and /u/, each with tense and lax allophonic variants. It thus differs from Mandarin, the focus of the Leong et al. (2021) study. Front /i/ is produced as [i] in open syllables, and before voiceless labials and alveolars, but as [I] before voiceless velars (Zee, 1991; Chan and Li, 2000). For /u/ the parallel lax variant ([℧]) also occurs before velars. Voiced obstruents do not occur in syllable-final positions. Additionally, [I] and [℧] are relatively lowered (Zee, 1991). Taken together, these facts indicate that the English rhymes /it/, /ut/, /Ik/, and /℧k/ have rough “matches” in Cantonese, but /It/, /℧t/, /ik/, and /uk/, along with all V+/d/ combinations, do not.

A simple transfer-based analysis would predict that the matching English rhymes should be easier for Cantonese speakers to acquire than the non-matching ones. However, Munro (2021) found extensive evidence to the contrary. First, Cantonese speakers' productions of several non-matching rhymes were, on average, more intelligible than those of the matching rhymes. That outcome was not particularly surprising, since the degree of “match” between L1 and L2 rhymes was by no means exact. Second, the speakers differed from one another in their success in producing the English tense-lax distinction. More intriguingly, they also differed in their success in producing identical VC rhymes in different words. For instance, some speakers produced the vowel in *sit* with high intelligibility, but not the vowel in *hit*; others showed the reverse pattern: high intelligibility for *hit* but not for *sit*. Still others produced both words with full intelligibility, and a fourth subset produced neither word intelligibly. Given the occurrence of all four patterns, the differences between words cannot be attributable to an effect of different initial consonants. An understanding of results such as these requires a close examination of how individuals vary from one another in their production capabilities.

As noted by a reviewer, an additional complication in the interpretation of the Munro (2021) results is that the speakers may have been exposed, to varying degrees, to HKE. The HKE vowel system is described by Hung (2000) as having seven phonemic monophthongs, with a neutralization of the high tense-lax distinction. As a result, speaker-participants may have heard words such as *hit* and *wood* modeled with tense vowels rather than the lax counterparts used in WCE. In such cases, not making a tense-lax distinction could be due partly or mainly to exposure to particular native HKE productions, rather than to L1–L2 transfer.

#### Design

In the previously published study, 18 Cantonese speakers produced multiple tokens of 31 English target words in a picture elicitation task. Targets were common real words with segmental VC combinations known to pose difficulty for Cantonese speakers. Some of the combinations approximately matched sequences occurring in Cantonese (e.g., /it/ and /Ik/), while others did not (e.g., /It/ and /ik/). Picture naming was used instead of word reading for two main reasons. First, it verified the speaker's knowledge of the target words and required speakers to access stored phonological knowledge in order to produce them. Second, it was expected to reduce orthographic influences that might be more evident in a reading task. Such effects might be particularly noticeable for the orthographically opaque contrast between /u/ (too, moon, boot) and /℧/ (took, look, book). Intelligibility was selected as the independent measure because of its status as the single most important index of communicative ability (Subtelny, 1977; Munro, 2011). Because this dimension of speech cannot be assessed directly with acoustic measures, judgments from trained listeners were obtained.

In this extension of the earlier work, an interrupted repetition task was added, in which speakers first heard an aural exemplar of the target word and were required to count aloud to 10 before producing it. This task was selected instead of immediate repetition so as to minimize the speaker's opportunity to access or “play back” an acoustic image from the short-term auditory store. Like the priming task used by Leong et al. (2021), the speaker heard a good exemplar of the target vowel prior to production. However, in this case, the entire target word was the stimulus, and no other means of elicitation were used (e.g., no on-screen orthographic presentation). It was expected that speakers would have to process the auditory input and recognize the word in order to recall it after counting. Doing so could facilitate the activation of perceptual representations which may be more difficult to access during picture naming without an aural prompt. Past work (Trofimovich and Gatbonton, 2006) indicates that priming effects can persist even after a distractor task. If so, then words elicited *via* interrupted repetition could be expected to have more intelligible vowels than those elicited *via* picture naming. This might indicate that L2 speakers have more phonological knowledge–developed through perceptual experience–than they are necessarily able to exploit when producing L2 segments without an aural model.

#### Research Questions

A key research question arose from the practical issue of how elicitation methods affect L2 vowel productions: (1) Does (high) vowel intelligibility differ in word productions elicited with and without a preceding auditory model? A second question follows from the finding of large variability in the intelligibility of vowels elicited *via* picture naming in Munro (2021): (2) To what degree is the effect of the elicitation task consistent across targets and across individual speakers?

## Methods

### Speakers

The L2 speakers (10 female; 8 male)–the same as those in Munro (2021) –were 18 Cantonese-speaking adults (M_age_ = 18 years; range: 15–25), who had been born and raised in Hong Kong and were residing in Canada at the time of the investigation [Mean length of residence (M_LOR_) = 4.9 years; range = 0.75–6.9; Mean age of arrival in Canada (M_AOA_) = 18 years; range = 15–25 years]. They were recruited *via* email and word-of-mouth, with the requirement that they self-identify as second-language speakers of English. All had grown up speaking Cantonese at home and all had studied English in grade school. However, none reported regular use of English for social purposes before immigration to Canada. More than half (*n* = 11) had received some ESL instruction in Canada on arrival. At the time of the study, all participants' English skills were advanced enough for them to be studying at English-speaking post-secondary institutions. On average, they reported using English 26% of the time in their day-to-day activities. For comparative purposes in the intelligibility assessment, recordings from two native speakers (1 female; 1 male) of General Canadian English (GCE) were also randomly selected from a database of speakers from a post-secondary student cohort. All speakers passed a pure-tone hearing screen (250–4,000 Hz) at 20dB_HL_.

### Speech Materials

The stimulus items, identical to those used by Munro (2021), were common English CV(C) words representing rhymes with and without “matching” analogs in Cantonese, as shown in Table 1. The particular targets were selected because they were likely to be known to the speakers, because they represented VC combinations either corresponding or not corresponding to sequences occurring in Cantonese, and because they could easily be represented visually for elicitation in a picture-naming task. Although several minimal pairs were included, the latter requirement made it impossible to create a fully balanced set.

**Table 1:** Stimulus items according to syllable structure.

**Vowel**	**Coda**	**Target words**	**“Matching” rhyme in Cantonese**
/i/	#	*key, see, tea*	Yes
	/t/	*feet*, heat, *seat*	Yes
	/k/	*cheek, speak*	No
	/d/	*feed, read*	No
/I/	/t/	hit, sit	No
	/k/	chick, kick, sick	Yes
	/d/	kid, lid	No
/u/	#	*Sue, two*	Yes
	/t/	boot, suit	Yes
	/k/	*Luke, tuque*	No
	/d/	food	No
/℧/	/t/	foot, put	No
	/k/	book, cook, look	Yes
	/d/	*good, wood*	No

*Italicized words were excluded from the analyses because intelligibility for those items in PNam exceeded 80%*.

### Previous Picture Naming Task (PNam)

Details of the PNam task were reported in Munro (2021). The speakers viewed a randomized set of drawings presented individually on letter-size cards, each displaying a stimulus number and the first letter of the target word as a clue. During a practice and familiarization session, a research assistant presented the entire set of cards one at a time as the speaker guessed the target and produced the stimulus number and the target as follows: “Number __. The next word is __.” When the guess was wrong, the speaker was instructed to “try again” and to make as many further attempts as necessary until the correct item was named. After the practice round, the assistant shuffled the cards and recorded productions of the full set. This step was repeated twice for a total of three recorded productions of each item. In case of any false starts or hesitations on the part of the speaker (<1% of cases), a further repetition was elicited. Distractor items were included at the beginning and end of each round to minimize the effects of list intonation. Inclusion of the stimulus numbers in the recordings facilitated later sorting and digital extraction of the stimulus words.

### Interrupted Repetition Task (IntRep)

In the IntRep task, the stimuli – identical to those in PNam – were presented aurally in the frame “The next word is ___,” *via* an audio recording produced by a male native speaker of GCE. As in PNam, items were randomized and presented in three rounds. The speakers were instructed to listen to the model sentence, count to ten orally, and then reformulate the model as “Now I say ___.” A short (2-min) practice round was provided immediately before the first round of recording. The stimuli were presented *via* custom playback software, controlled by a research assistant, who monitored the performance of each speaker. In the event, that a production was missed or otherwise unusable (e.g., a false start or hesitation), the stimulus was replayed (< 1% of cases).

### Recording Procedures

During the individual sessions, high-quality digital recordings (44.1 kHz; 16 bits quantization) were made in a sound-treated booth. Speakers wore a Shure Beta 54 head-mounted microphone connected to a Symmetrix 302 microphone preamplifier and an HHB Professional digital recorder (CDR-830). They completed the speaking tasks with PNam preceding IntRep so that they were not exposed to aural models of the target words prior to PNam. As a result of this ordering, in advance of IntRep, the participants were fully familiar with the words, having produced them four times each in PNam, once during a practice round and three times during recording. A break between tasks was given for as long as each speaker desired (typically about 5 min), and drinking water was available as needed.

### Listener-Judges

The same four linguistically trained assistants who had evaluated vowel intelligibility in Munro (2021) also judged the IntRep productions in this study. All judges had grown up in Canada in monolingual (Canadian English) households. Two had taught ESL extensively, one had studied Japanese and Korean, and the fourth had extensive experience listening to and measuring non-native speech as a lab assistant. All were familiar with IPA, and all passed the pure-tone hearing screen referenced above.

### Token Extraction and Intelligibility Evaluation

After recording, the target word productions were digitally excised and saved as individual peak-normalized audio files. These were evaluated for intelligibility by the listener judges during multiple individual sessions held over several days. Each judge heard a different randomized presentation of the excised words through high-quality headphones and, on a computer screen, selected the symbol for the GCE vowel closest to the one they heard in each production. The available response choices were based on pre-screening of the tokens by the author so as to include vowels that were not actual targets, but were sometimes produced in error: / i I eI ε u ℧ o℧ /. Inter-judge reliability was assessed on the basis of whether the judge assessed a production as on-target or not. Four-way agreement was found on 72% of items, with at least three of four judges agreeing on 92%. These rates are slightly higher than for Munro (2021) and compare favorably with rates in other L2 vowel studies (Munro and Derwing, 2008).

## Results

In Munro (2021), the PNam data were submitted to multiple analyses, including comparisons of mean performance on vowels, rhymes, and words. Because the present study has a more restricted focus than the earlier one–i.e., the effect of elicitation type on vowel intelligibility–the statistical analyses were selected so as to focus on questions relating specifically to that issue. The earlier study, which incorporated self-estimated English use and length of Canadian residence as co-variates, yielded non-significant effects of each, so these were not included here.

### Preliminary Analysis

To begin, exploratory probing of the data was carried out to identify suitable directions for more detailed analyses. First, mean intelligibility scores for the two tasks were computed for each speaker by pooling the scores of the four judges over the 31 words for each speaker. A paired samples *t*-test indicated significantly higher scores for IntRep (M = 79.4, SE = 1.78) than for PNam (M = 72.9, SE = 1.99), *t* (17) = 5.265, *p* < 0.001, *d* = 1.241). The differences between task scores varied considerably across speakers. The maximum difference was 16 percentage points; however, two speakers showed a difference of only 1 point, and two others showed a small reversal, with PNam > IntRep by 2 and 3 percentage points.

Second, because IntRep always followed PNam, it might be proposed that the better performance on IntRep could simply be due to a greater amount of practice with the target items. If so, then one would expect intelligibility to increase over successive recorded trials for one or both tasks. Repeated measures ANOVAs on the intelligibility scores revealed no significant effects of Trial Number for either PNam, *F* (2, 34) = 0.515, *p* = 0.602 or IntRep, *F* (2, 34) = 0.024, *p* = 0.977. The fact that there was no evidence of improvement over time *within* tasks suggests that the between-task difference was likely due to the task itself rather than to a practice effect.

Next, a two-factor repeated measures ANOVA yielded significant effects of both Vowel, *F* (1.451, 24.67) = 45.669, *p* < 0.001(Greenhouse-Geisser, due to a violation of the sphericity assumption), $\eta_{p}^{2}$= 0.729 and Task *F* (1, 17) = 29.381, *p* < 0.001, $\eta_{p}^{2}$ = 0.633. Relevant means are given in Table 2. The interaction of Vowel and Task missed significance, *F* (2.085, 35.44) = 2.344, *p* < 0.109 (Greenhouse-Geisser), $\eta_{p}^{2}$ = 0.121. Despite the lack of significance, the latter effect size falls between medium and large (Cohen, 1988). At the request of a reviewer, coefficients of variation, which can be understood as “standardized” measures of variability are provided in Table 2. In general, these are considerably smaller for the tense vowels, suggesting greater precision of estimation of population means for those vowels. However, because of complications arising from inter-speaker differences to be discussed later, it is inadvisable to dwell at length on these outcomes.

**Table 2:** Mean intelligibility by vowel and task (all targets).

**Vowel**	**PNam mean (%)**	**SD**	**COV**	**IntRep mean (%)**	**SD**	**COV**	**COV diff ratio (%)**
i	92.3	9.72	0.105	94.4	6.42	0.068	35
I	50.7	22.67	0.447	59.1	18.87	0.319	29
u	82.2	12.26	0.149	93.1	7.56	0.081	46
℧	58.3	11.56	0.198	65	15.21	0.315	−18

An examination of vowel confusion data from the listeners revealed similar patterns to those seen in the study by Munro (2021) in that non-native-like lax targets were not always produced as their tense counterparts. On the one hand, 5% of /i/ targets were judged to be /I/ with only 1% heard as something else. On the other, for /I/ targets, 20% were heard as /i/, 18% as /e/, and 3% as others. Back, tense /u/ was heard as /℧/ in 5% of cases, and as /o/ or others in 2%, while /℧/ was heard as /u/ 11% of the time and as /o/ 23% of the time.

Finally, it was determined that some stimulus words did not need to be included in the statistical analyses because the performance was at or near the ceiling in the PNam task. In fact, there was essentially no inter-speaker variability in many of the target items due to 100% accuracy. Given the preliminary analysis above, it was expected that scores on IntRep would also be at or near the ceiling for those items such that the inclusion of these data would grossly violate distributional assumptions for statistical modeling. The criterion for exclusion of words was set at a mean (across all speakers) of 80% or greater intelligibility on the PNam task, the same criterion used by Munro (2021) as an indicator that a particular L2 rhyme had been “acquired.” On that basis, 15 words were excluded. For the excluded items, the mean intelligibility difference between tasks (IntRep%–PNam%) was relatively small, only +2.96 percentage points, compared with +10.4 points for the included words. For two excluded words, the IntRep score was slightly lower than the PNam score (−0.9 points for *seat* and −2.3 points for *wood*). Means determined by Task and Word are discussed in a later section. The group-based analyses that follow are based on items remaining after the exclusion, which will be referred to as the “included words.” It should be noted that IntRep scores on five included words exceeded 80%. This was not expected to pose problems for the analyses because of considerable interspeaker variability on those items.

### Statistical Modeling

Scores for the included words were evaluated with mixed-effects models in *JASP* (JASP Team, 2022), in which Speaker was treated as a random effect. Because some of the fixed effects were not independent, three separate models for fixed factors (Rhyme, Word, and Matching Status) had to be computed (Type III Sum of Squares, Kenward-Roger procedure). *Post-hoc* analyses were Bonferroni-adjusted *t*-tests. In all results, *p* < 0.05 was adopted as the level for significance.

#### Rhyme Analysis

In 13 of the 14 rhymes in the original PNam recordings, the mean intelligibility of all words associated with the rhyme was either consistently above (*n* = 6) or below (*n* = 7) the “acquired” criterion of 80%. In short, rhyming words tended to “behave” in the same way. In the case of /i/, however, two words reached the criterion (*feet* and *seat*), while the third (*heat*) did not. Because the inclusion of only one of the three items in the modeling would give a misleading picture of the task differences across rhymes, *heat* was omitted from the rhyme analysis, which was therefore based on the six rhymes in which no words reached the intelligibility criterion in PNam. For the mixed-effects ANOVA results in Table 3, Rhyme and Task were fixed factors. Including random slopes for speakers led to a singular fit, so these were not modeled. Both main effects reached significance, as did the two-way interaction. *Post-hoc* tests revealed that the Task effect was due to significantly higher vowel intelligibility on IntRep for /ud/ and /ut/, but the between-task differences for /Ik/, /It/, /℧k/ and /℧t/ were not significant despite a general trend toward higher intelligibility on IntRep. Means by rhyme and task are given in Table 4.

**Table 3:** Mixed effects ANOVA (rhyme and task) for included words.

Effect^*^	***df***	***F***	***p***
Rhyme	6, 509	26.322	<0.001
Task	1, 509	31.091	<0.001
Rhyme ^*^ task	6, 509	2.191	0.043

* *Speakers were entered as a random effect*.

**Table 4:** Mean intelligibility by rhyme and task (included rhymes only).

**Rhyme**	**PNam%**	**SE**	**95% CI**		**IntRep%**	**SE**	**95% CI**		**Between-task**
			**Lower**	**Upper**			**Lower**	**Upper**	**Difference**
/ut/	69.9	5.23	59.7	80.2	84.3	5.23	74	94.5	14.4^*^
/℧t/	68.5	5.23	58.3	78.7	78.8	5.23	68.5	89	10.3
/ud/	61.7	6.87	48.2	75.1	93.6	6.87	80.1	107	31.9^*^
/id/	55.3	5.23	45.1	65.6	65.1	5.23	54.8	75.3	9.8
/ik/	52.0	4.55	43.1	60.9	51.9	4.55	42.9	60.8	−0.2
/it/	44.1	5.23	33.9	54.4	63.9	5.23	53.7	74.1	19.8
/℧k/	33.2	4.55	24.3	42.1	41.2	4.55	32.3	50.1	8.0

* *p_bonf_ < 0.05*.

#### Word Analysis

Table 5 gives ANOVA results for the Word and Task analysis. Once again, including random slopes for speakers led to a singular fit, so these were removed. Both the Task and Word effects were significant, though the two-way interaction was not. The Task effect was due to higher scores on IntRep than on PNam, as seen for the pairs of means shown in Table 6, though one reversal (*kick*) was observed. Given the absence of a significant interaction, *post-hoc* tests were carried out on the combined results of the two tasks to compare scores on words sharing a rhyme. The results, shown in Table 7, are similar, but not identical to the results for PNam alone, reported by Munro (2021). In particular, different patterns were seen for /Ik/, in that for the combined results, the vowel of *sick* was significantly more intelligible than that of *kick* or *chick;* and for /℧k/, in that the *look* vowel was more intelligible than the *cook* vowel.

**Table 5:** Mixed effects ANOVA (word and task) for included words.

Effect^*^	***df***	***F***	***p***
Word	15, 527	16.772	<0.001
Task	1, 527	23.879	<0.001
Word ^*^ task	15, 527	1.270	0.216

* *Speakers were entered as a random effect*.

**Table 6:** Mean intelligibility by word and task (included words only).

**Word**	**PNam%**	**SE**	**95% CI**		**IntRep%**	**SE**	**95% CI**		**Between-task**
			**Lower**	**Upper**			**Lower**	**Upper**	Difference*
heat	73.2	0.93	60.3	86.2	75.4	0.80	62.5	88.3	2.2
hit	41.6	0.95	28.6	54.5	61.6	0.84	48.7	74.5	20.1
sit	46.7	1.09	33.8	59.7	66.2	0.88	53.2	79.1	19.4
chick	40.7	0.89	27.8	53.7	47.3	0.78	34.3	60.2	6.6
kick	53.7	0.84	40.8	66.7	40.7	0.61	27.8	53.7	−13
sick	61.6	0.66	48.7	74.5	67.6	0.30	54.6	80.5	5.9
kid	75.4	1.07	62.5	88.4	82.9	0.79	70	95.8	7.4
lid	35.2	1.11	22.2	48.1	47.3	1.10	34.3	60.2	12.1
boot	66.2	0.62	53.3	79.2	81.4	0.70	68.5	94.4	15.2
suit	73.6	0.66	60.7	86.5	87.1	0.42	74.2	100	13.5
food	61.7	0.90	48.7	74.6	93.6	0.26	80.6	106.5	31.9
foot	60.1	0.90	47.2	73	69.9	1.01	57.0	82.9	9.8
put	76.9	0.61	64	89.8	87.6	0.49	74.7	100.5	10.7
book	36.1	0.78	23.1	49	41.2	0.52	28.3	54.2	5.2
cook	26.3	0.71	13.3	39.2	33.4	0.71	20.5	46.3	7.1
look	37.2	0.79	24.2	50.1	48.9	0.63	36	61.9	11.8

* *IntRep% – PNam%*.

**Table 7:** *Post-hoc* comparisons^*^ of %-correct ID for words with identical rhymes.

**Rhyme**	**PNam (Munro, 2021)**	**PNam and IntRep combined**
/it/	hit = sit	hit = sit
/ik/	[sick = kick] > chick	sick > [kick = chick]
/ut/	boot = suit	boot = suit
/℧t/	put > foot	put > foot
/℧k/	book = cook = look	book = [look > cook]
/Id/	kid > lid	kid > lid

* *No difference shown by “=”; significant difference (p_bonf_ < 0.05) shown by “>”*.

#### Matching Status Analysis of Rhymes

**Figure 1:**
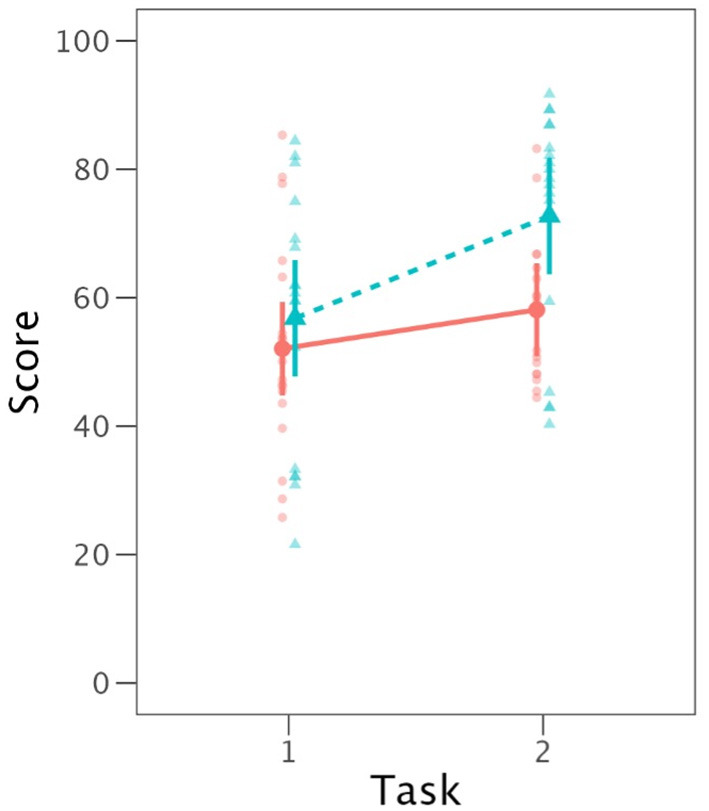
Mean vowel intelligibility (with 95% CI) according to task (1 = PNam, 2 = IntRep) and matching status (solid line = match, dotted line = no match).

Results of the third analysis, in which Matching Status (of rhymes) and Task were fixed effects, are given in Table 8. In this case, by-speaker random slopes were included for each matching condition. The effects of both Matching Status and Task were significant, with the two-way interaction marginally so. Figure 1 illustrates these outcomes. *Post-hoc* analyses indicated that for IntRep, non-matching items were produced with higher intelligibility than matching ones. For PNam, no statistical difference between matching and non-matching items was observed.

**Table 8:** Mixed effects ANOVA (matching status and task) for included words.

Effect^*^	***df***	***F***	***p***
Matching status	1, 17	5.923	0.026
Task	1, 538	19.915	<0.001
Matching status ^*^ task	1, 538	4.024	0.045

* *Speakers were entered as a random effect*.

### Individual Speaker Performance

**Figure 2:**
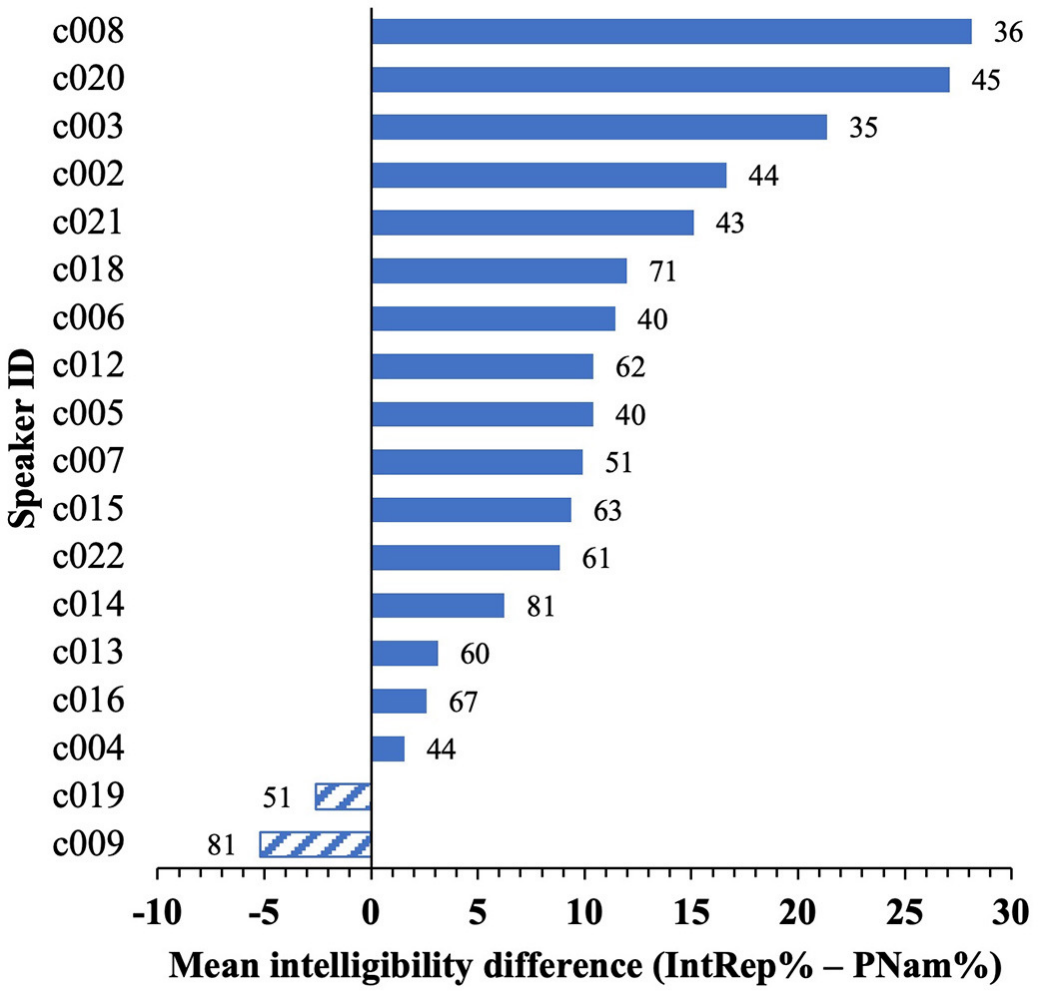
By-speaker differences between mean intelligibility scores on the two tasks for the 16 included items. Data labels are the PNam% scores.

As noted earlier, speakers varied in the degree to which their vowel intelligibility differed across tasks. Intelligibility differences according to speaker are given in Figure 2 (IntRep%–PNam%). Sixteen speakers showed higher mean scores for IntRep, with a difference of 2–28 percentage points, while 2 showed marginally lower scores (≤5 points). There was a small-to-moderate negative correlation (assessed non-parametrically because of uneven data distributions and small sample size) between the differences and the corresponding PNam scores (Spearman's *rho* = −0.503, *p* < 0.033), indicating that speakers with overall lower PNam scores tended to show greater differences on IntRep. This finding is unsurprising, since a lower score on PNam indicates “more room to improve” on the subsequent task. Despite these noteworthy differences between speakers, the mean intelligibility for individual speakers on PNam showed a moderately high correlation with scores on IntRep, *r* (16) = 0.8, *p* < 0.001, indicating that PNam scores predicted IntRep scores rather well (See the Supplementary Materials for scatterplots showing both patterns reported above). In response to a reviewer query, I stress that my use of the term “improvement” here and elsewhere refers only to the between-task difference. I am not assuming any sort of “learning” or permanent change in performance as a result of the simple exposure provided in IntRep.

#### Individual Task Effects by Word

To extend Munro's (2021) examination of differences among speakers on words with the same rhyme, the task effect was explored for individual speakers through visual inspection of descriptive data on each word, along with figures comparing speakers. Because the nature of the data did not allow inferential statistics, a conservative criterion of a change of more than ±25 percentage points was adopted to assess whether a between-task difference on a particular target was of importance. Selected cases will be highlighted here because of the interesting findings they illustrate. For completeness, the figures for the full set of included words are provided in the Supplementary Material.

In 22% of the 288 word-by-speaker combinations (18 speakers and 16 words), intelligibility improved by more than 25 points in IntRep. In 6%, it dropped by more than 25 points, with the remaining majority (72%) showing <25 points difference.

**Figure 3:**
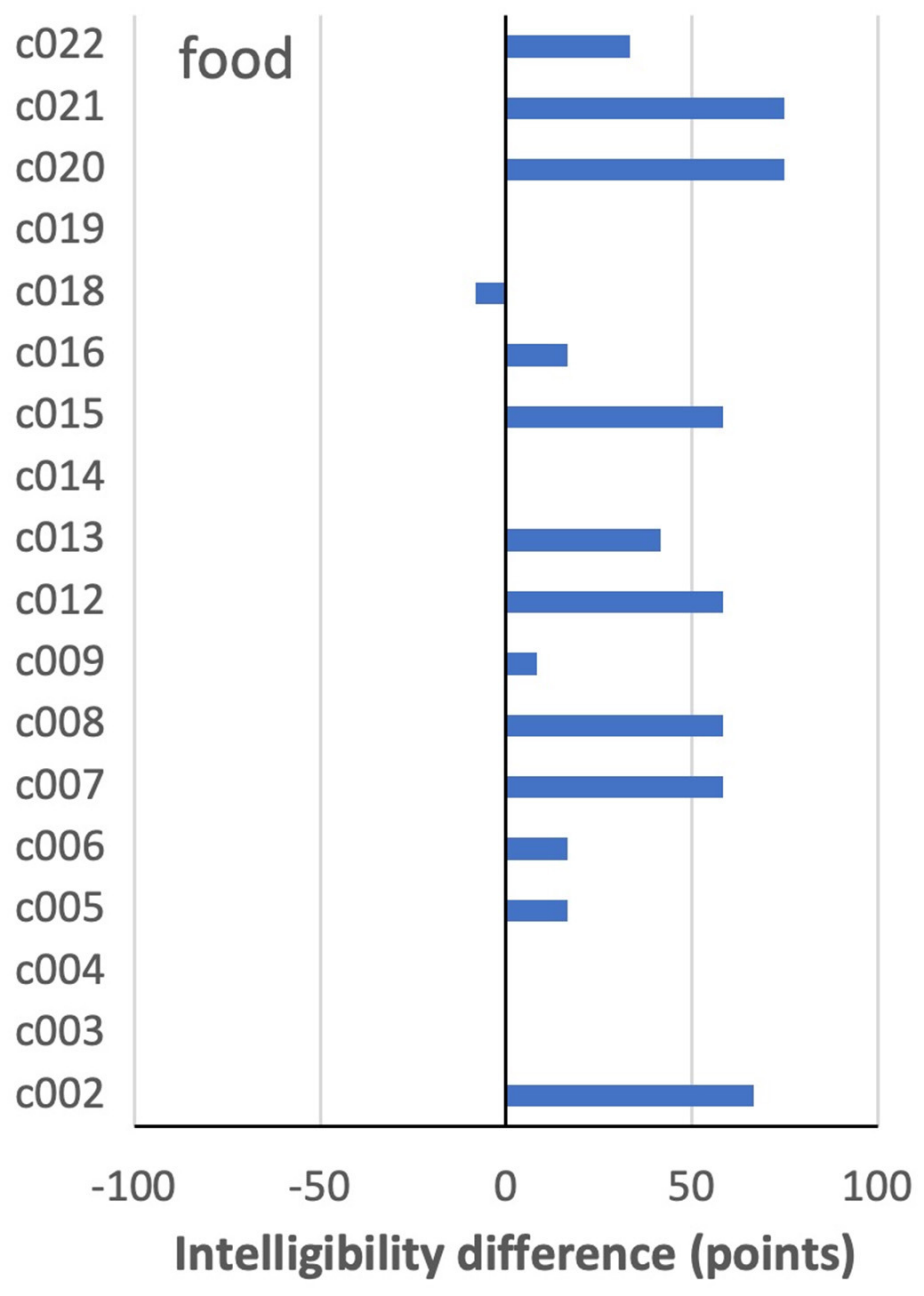
By-speaker differences between mean intelligibility scores on the two tasks for *food*.

The word showing the largest overall improvement on IntRep was *food* (62 vs. 94% intelligibility). As shown in Figure 3, nine speakers showed a difference of more than +25 points on IntRep. Of the remaining speakers, who showed a smaller between-task difference (<25 points), all but one scored above 83% on PNam, indicating a high level of performance and, therefore, little room for improvement. Thus, in the sense that nearly all who could potentially show a task effect did so, *food* might be regarded as showing a relatively consistent effect across speakers.

**Figure 4:**
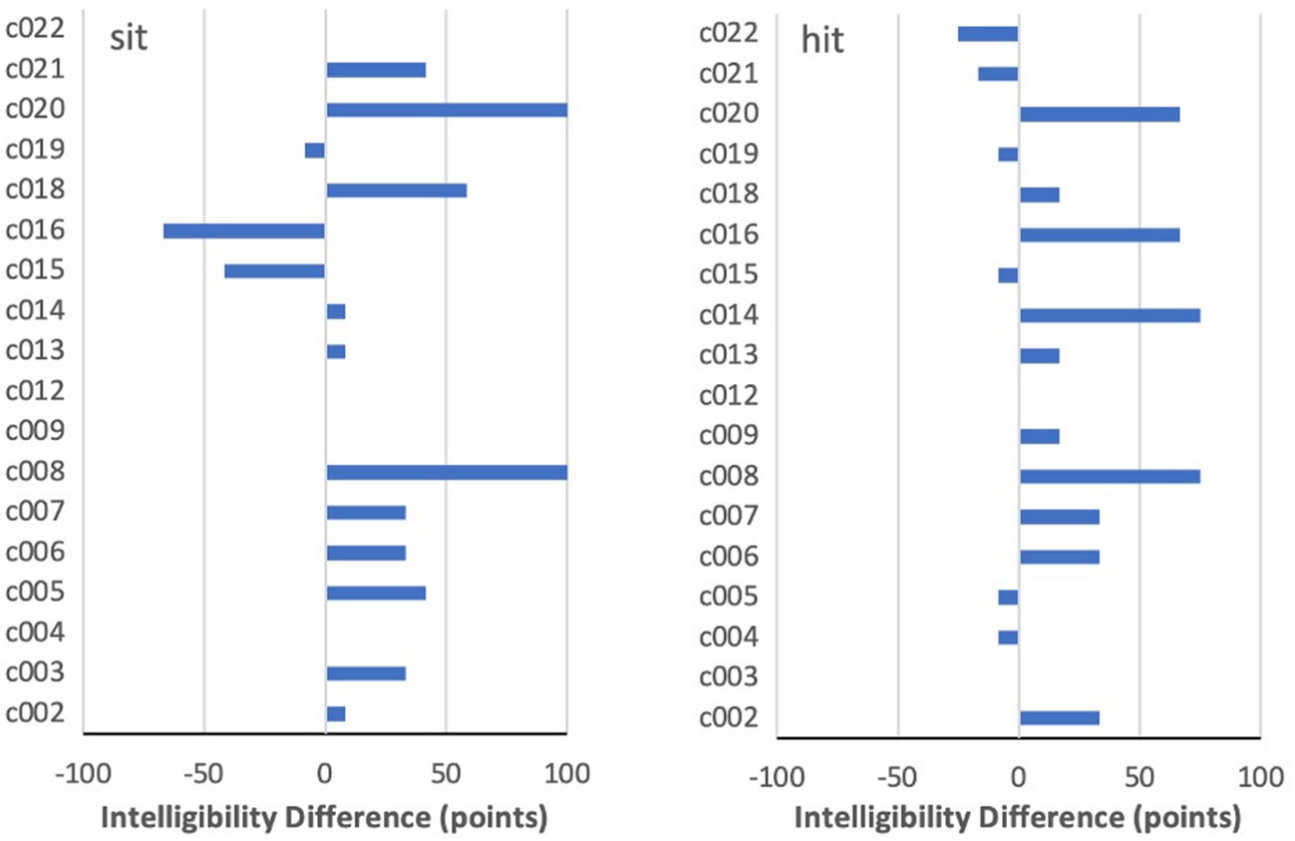
By-speaker differences between mean intelligibility scores on the two tasks for *sit* and *hit*.

For most other target words, however, there was much less consistency. Two interesting differences between PNam and IntRep are seen for *sit* in Figure 4, where c008 and c020 showed a complete change from 0 to 100% intelligibility. The same two speakers had also performed near 0% on *hit* in PNam, and, while both improved on that word in IntRep, the change was somewhat smaller (75 points and 67 points). In contrast, c004 and c019, who also performed at <10% on *sit* in PNam, showed virtually no task difference on *sit*. Moreover, among all speakers, improvement of 25 points or more on either *sit* or *hit* did not necessarily entail any improvement on the other word in the pair. This was true for c021, who improved on *sit* from 33 to 75%, but had surpassed the 80% threshold on *hit* in PNam. Particularly striking is c016, who improved by 67 points on *hit*, but declined by an equal amount on *sit*. Although four of the six speakers (c009, 12, 14, and 22) who performed above 80% on *sit* in PNam showed no meaningful difference between tasks, the two others (c015, 16) declined noticeably in IntRep.

**Figure 5:**
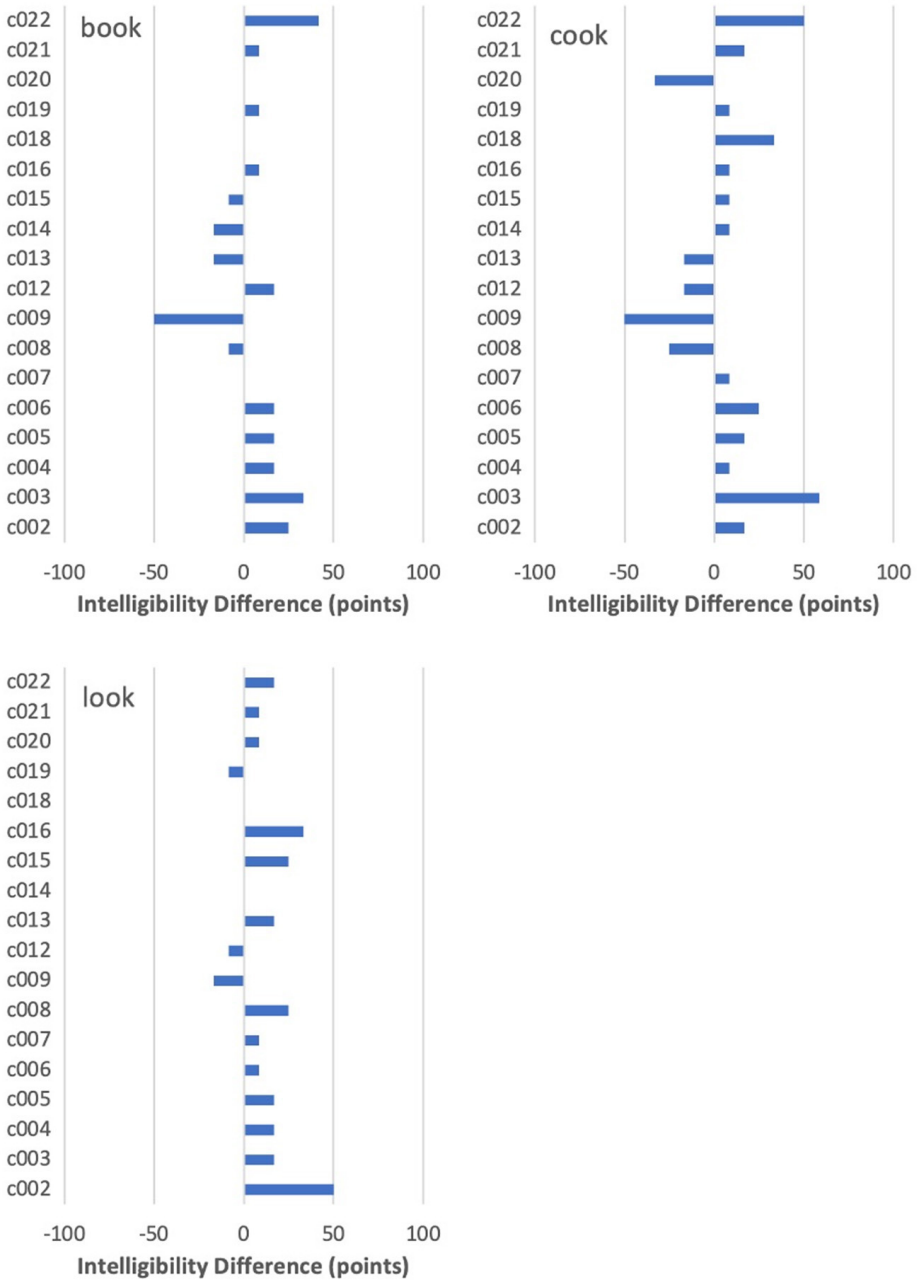
By-speaker differences between mean intelligibility scores on the two tasks for *book, cook*, and *look*.

Differences among the three /℧k/ words are shown in Figure 5. These contrast to varying degrees with the comparatively consistent pattern for *food*. In general, the /℧k/ targets ranked very low on intelligibility in PNam, with only two speakers scoring above 80% on *book*, two on *look*, and not a single speaker on *cook*. For the latter item, three speakers (PNam scores of 8, 50, and 17%) showed an increase of more than 25 points on IntRep, with most of the remainder showing little change. Given that seven of the non-changers had scored <25% on PNam, it is clear that “having room” to improve across tasks was not a good indicator that such improvement actually would occur. Two reversals of more than 25 points occurred for *cook*, with one for *book* and none for *look*. It is also worth noting that c016, who showed an increase of 33 points on *look*, showed a negligible difference on *cook*, despite having plenty of room to improve on that item from a PNam score of only 16%.

**Figure 6:**
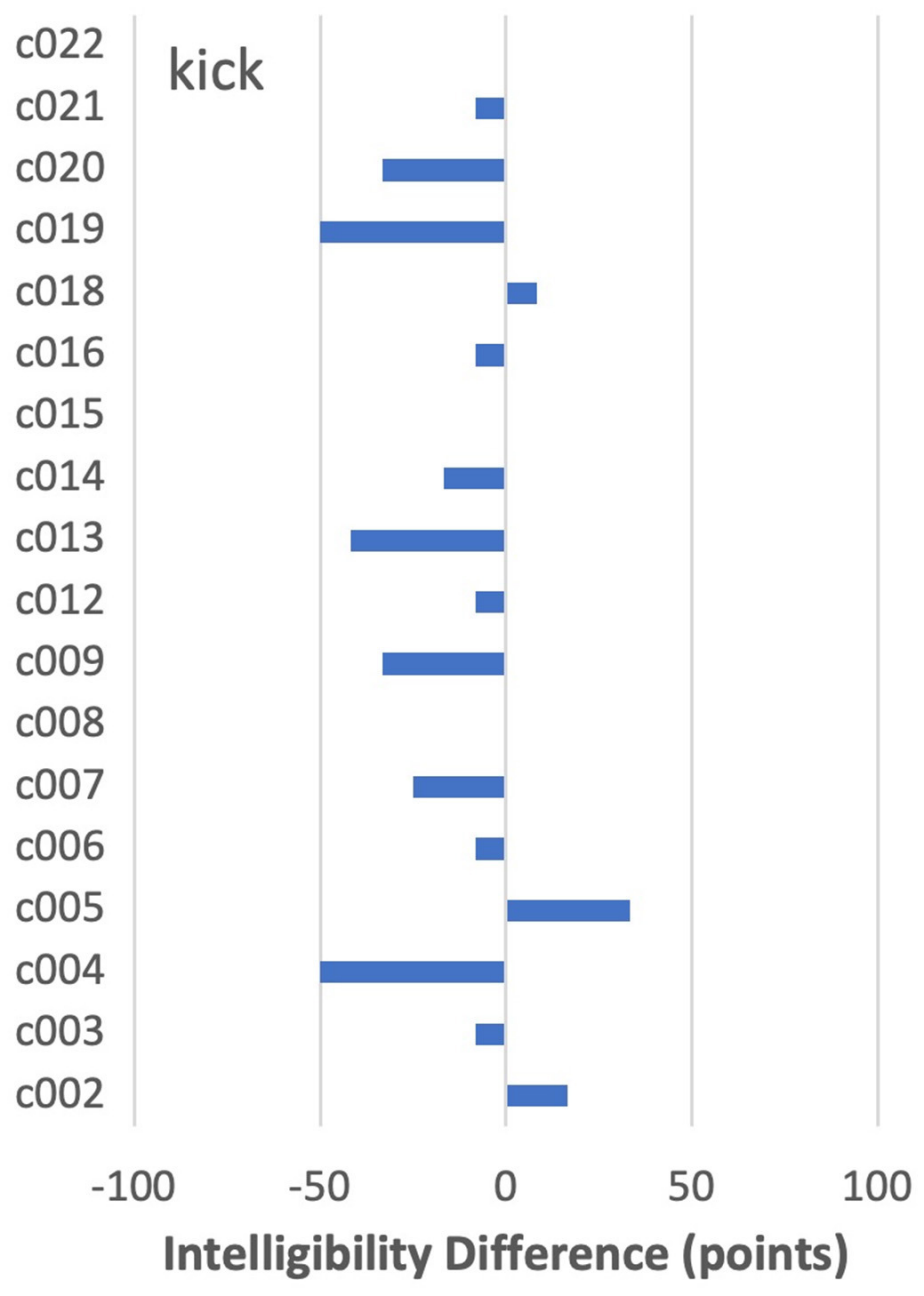
By-speaker differences between mean intelligibility scores on the two tasks for *kick*.

As noted earlier, in about 6% of word-by-speaker combinations, a decline in the intelligibility of more than 25 points was seen from PNam to IntRep. For the most part, these cases appeared to be randomly distributed across words, with one or two cases per word. The only exception was *kick*, shown in Figure 6. For that item, only one speaker (c005) improved by more than 25 points, with five speakers showing declines of at least that magnitude.

One final illustration of the inconsistency of the task effect can be seen in a comparison between c002 and c004, both of whom scored 44% on PNam and therefore fell into the lower half of the speaker cohort. For c002, there was an overall improvement on IntRep of 17 points, with increases of more than 25 points on three words (*hit, food, look*) and declines on none. In contrast, c004 had a net improvement of only 2 points, with an improvement of more than 25 points on only one word (*foot*), and a comparable decline on two words (*kick, boot*).

## Discussion

This study was designed to address two questions relevant to the production of English high vowels by Cantonese L2 speakers: (1) whether vowel intelligibility would differ on two elicitation tasks and (2) whether any observed task effect would be consistent across different production types and across different speakers. In general, the first question can be answered in the affirmative, which means intelligibility on the interrupted repetition task (IntRep) was higher by more than 10 percentage points than on picture naming (PNam). As for the second question, however, substantial evidence of several types of inconsistency was obtained. Consequently, the finding of an intelligibility benefit for IntRep must be interpreted with caution.

### Why a Task Difference?

Before addressing these inconsistencies, it is appropriate to ask why the IntRep task should offer an intelligibility benefit to begin with. Findings indicate that in Munro (2021) and in the current study, vowel intelligibility was somewhat tied to lexical knowledge. Considering first the PNam task, note that some speakers produced certain targets with an intelligible vowel, but not other targets, even those sharing the same rhyme. Some speakers showed opposite patterns to other speakers, so that one speaker produced the *hit* vowel accurately but not the *sit* vowel, while another performed correctly on *sit*, but not on *hit*. To account for these inconsistencies, it is not possible to appeal exclusively to speakers' knowledge of vowels or rhyme-size units. Rather, the speakers evidently had knowledge of the motoric programs needed to produce /℧/ and /℧k/ which they sometimes employed, but which did not necessarily transfer to all situations. In these cases, many inconsistencies appear to have been due to non-native-like lexical representations the speakers had developed, as in the study by Llompart and Reinisch (2019). However, the results of the PNam task gave no firm indication of how speakers would perform when given support in the form of an aural production of the target. Multiple explanations might be offered for why IntRep sometimes yielded better intelligibility. While this study was not designed to provide a definitive account, one possibility is that speakers sometimes had established more than one lexical representation for a word: one non-native-like, perhaps developed early in the acquisition process, and a competing, but more weakly established, representation developed through repeated exposure to a native or native-like target. A correct production might occur in IntRep if the aural prompt (a good exemplar) activated the native-like representation to a greater extent than the incorrect one. This is not implausible. Even native speakers, for instance, sometimes appear to have more than one representation for certain lexical items. Some L1 English speakers might, for instance, establish a non-standard lexical representation for *epitome* based on a “reading pronunciation,” viz /'εpɘto℧m/, and use that pronunciation when speaking extemporaneously. However, they may simultaneously hold another representation developed from hearing the word spoken aloud (/I'pItɘmi/) without awareness that the two representations correspond to a single word. Likewise, the availability of two different representations for *either*, one beginning with /i/, the other with /aI/), may result in varying pronunciations by the same speaker from one time to another. An alternative account is that the Cantonese speakers had only a single non-native-like lexical representation for a word, but the presentation of an aural model activated motoric programs for the correct rhyme in the target word, allowing the speaker to bypass the stored knowledge that was accessed during picture naming. Irrespective of the reasons for the task effect, the results suggest that PNam did not fully capture speakers' production capabilities. They often performed better when support was available in the form of an aural model. As discussed further below, that outcome may be relevant to interactive speaking contexts.

### Variability in the Task Effect

One outcome of the study was that scores on 15 of the 16 target words were high (>80%) on both tasks. These items could therefore be classified as “easy” for the L2 speakers, and the importance of any observed difference in scores between tasks would be doubtful. For that reason, there was no reason to examine the production scores in detail, and they were excluded from most statistical analyses. They confirm a conjecture raised in the introduction that some targets would show relatively uniform patterns across speakers (in this case, uniform ease) while others would not.

For the remaining words, several types of variabilities were observed in the task effect. In the first place, the magnitude of the task-related difference was not uniform across different rhymes. For instance, the rhymes /ud/ and /ut/, both containing the tense back vowel, were produced more intelligibly, on average, in the IntRep condition than in PNam, but the same was not true for the rhymes with the lax vowels, /Id/, /Ik/, /It/, /℧k/, and /℧t/. This was not simply the result of a ceiling effect due to high levels of performance for PNam on the lax vowel rhymes, as is clear from the means in Table 4, which in all but one case fell below the means for the tense vowels. But even the finding of differences between rhymes requires qualification because, as explained below, it did not generalize across speakers.

Second, “matching” vs. “non-matching” between L1 and L2 VC rhymes was tied to different outcomes. In particular, the significant interaction between matching status and rhymes occurred because English rhymes with no analog in Cantonese were produced more intelligibly than matching rhymes *only* in the IntRep condition.

Third, some speakers showed much higher mean intelligibility on IntRep than on PNam–more than a 25-point difference–while many showed little or no difference between the two. Thus, it is clearly not true that all speakers showed a net benefit from the presentation of a spoken model prior to production. Although there was no indication that any speaker performed meaningfully worse on IntRep than on PNam, some word-by-speaker combinations did show sizeable declines, as will be discussed further below.

Fourth, although no significant word-by-task effect emerged, that outcome, which is based on group means, hides interesting between-speaker differences. One word (*food*) showed relative consistency across speakers in that virtually all speakers who had not approached the ceiling in PNam did show increased scores in IntRep; however, that consistency was not common among the target items. Overall, fewer than 25% of word-by-speaker combinations showed an increase of 25 percentage points or more. Not only did the magnitude of the task difference vary from speaker to speaker, but, it also varied on different test items for different speakers. For instance, speakers c008 and c004 both performed poorly on *sit* in PNam, but c008 improved to perfect performance in IntRep, while c004 showed no change at all. Although it is tempting to speculate about why these two speakers patterned differently, no firm conclusions can be drawn. Both speakers had enrolled in ESL classes on arrival in Canada and both reported low daily use of English (<10%). It may be relevant that c008 had been in Canada for somewhat longer than c004 (5.6 vs. 3.8 years for c004). However, LOR did not prove to be a successful predictor of performance in the study by Munro (2021). It was also noteworthy that individual speakers who improved on a particular word (e.g., *look*) did not necessarily improve on other items with the same VC rhyme (e.g., *cook*). This makes it unlikely that any demographic variable such as LOR or L2 use can serve as a straightforward predictor of success in production.

Finally, of the 288 word-by-speaker combinations, about 6% showed a decline of more than 25 points on IntRep. These generally occurred at a rate of once or twice per word, with other smaller declines being more common. In some cases, these reversals might simply reflect random variability in production commonly referred to as regression to the mean. However, an unusually high number of them (5) was observed for *kick*. One possibility is that the nature of the model stimulus played a role. Although model stimuli were screened for categorical accuracy by a research assistant, it is conceivable that, for unknown reasons, some speakers who produced a correct target in PNam did not correctly recognize the model word during the IntRep task and therefore did not match it to their (correct) lexical representation of *kick*.

## Conclusion

Central to this research and to the study by Munro (2021) is the complex individual differences that emerged in the vowel intelligibility data. Although some individual variability in speech production is always attributable to “noise,” many of the patterns seen here are at least partially systematic, and cannot be dismissed as uninteresting simply because no immediate explanation is available. Rather, it is essential to closely examine the nature of this variation to determine what insights it may yield into the L2 speech learning process. In fact, there is a growing awareness of the value of studying individual learning trajectories in other aspects of L2 speaking as evidenced by the increasingly high profile of Complex Dynamic Systems Theory in L2 research (Lowie and Verspoor, 2022).

The findings reported here are likely to be of interest to assessors, language teachers, and researchers because they show that the choice of elicitation method can affect the intelligibility of L2 speakers' vowel productions. On the one hand, picture naming may be a useful way of determining speakers' typical pronunciations in unaided situations. On the other, the outcome of such a task may not capture the full knowledge a speaker possesses about pronouncing target items. The availability of an auditory model, however, seems to facilitate speaker access to knowledge that is not activated in picture naming. The between-task difference in performance raises interesting questions about the benefits of speech learning of interactions in which an L2 speaker hears the productions of an interlocutor and responds using some of the same lexical items as the interlocutor. In such circumstances, the modeled pronunciation may serve a scaffolding function that can facilitate more accurate production by the listener. Although it is possible that this modeling can promote additional learning, the degree to which such learning (if any) actually occurs has not been assessed here and is a topic worthy of further investigation. A more detailed examination of the kinds of effects observed in this study may lead to enhancements of models of L2 speech production (Kormos, 2014) and acquisition (Flege and Bohn, 2021). For example, it may prove useful to probe acoustic data from L2 speakers' productions in IntRep and other types of repetition tasks to determine the degree to which phonetic convergence toward the pronunciation of the model speaker occurs. It is noteworthy as well that the benefit of modeling apparently does not require *immediate* repetition by the speaker. That finding is consistent with research showing that priming effects can persist well after the presentation of the prime itself (Trofimovich and Gatbonton, 2006).

From the standpoint of phonetic learning research, a significant finding of Munro (2021) was that intelligible L2 production of a vowel in a particular word does not predict that the vowel will be produced correctly in other words, even those with the same post-vocalic environment. Rather, accurate pronunciation is somewhat linked to word learning. The present study adds a new complication to that finding in that the effect of presenting an aural model during elicitation is not uniform across targets or speakers. Although some speakers benefit considerably from such an approach, others do not, or they show the benefit on different targets. Consequently, accurate evaluation of segmental difficulties and strengths requires a more sophisticated approach than elicitation of a small number of target words representing the segments of interest.

## Data Availability Statement

The raw data supporting the conclusions of this article will be made available by the authors, without undue reservation.

## Ethics Statement

The studies involving human participants were reviewed and approved by Ethics, Simon Fraser University. The patients/participants provided their written informed consent to participate in this study.

## Author Contributions

The author confirms being the sole contributor of this work and has approved it for publication.

## Funding

This research was funded by a grant from the Social Sciences and Humanities Research Council of Canada. Open access publication was funded by Simon Fraser University.

## Conflict of Interest

The author declares that the research was conducted in the absence of any commercial or financial relationships that could be construed as a potential conflict of interest.

## Publisher's Note

All claims expressed in this article are solely those of the authors and do not necessarily represent those of their affiliated organizations, or those of the publisher, the editors and the reviewers. Any product that may be evaluated in this article, or claim that may be made by its manufacturer, is not guaranteed or endorsed by the publisher.
