# Supplementary material for: Variability in L2 Vowel Production: Different Elicitation Methods Affect Individual Speakers Differently
Source: Front Psychol. 2022 Jul 13;13:916736. doi: 10.3389/fpsyg.2022.916736 (PMC9326214; doi:10.3389/fpsyg.2022.916736)

Supplementary Figures

Figures comparing performance on each included word for the two tasks, with the difference (IntRep% - PNam%) on the x-axis and speaker code on the y-axis.

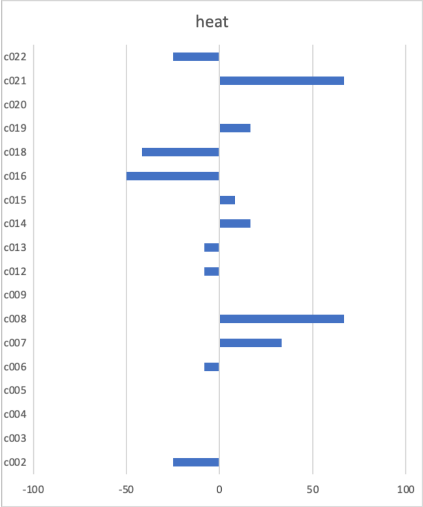

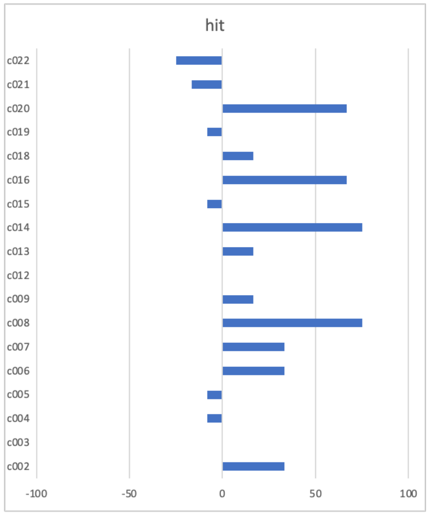

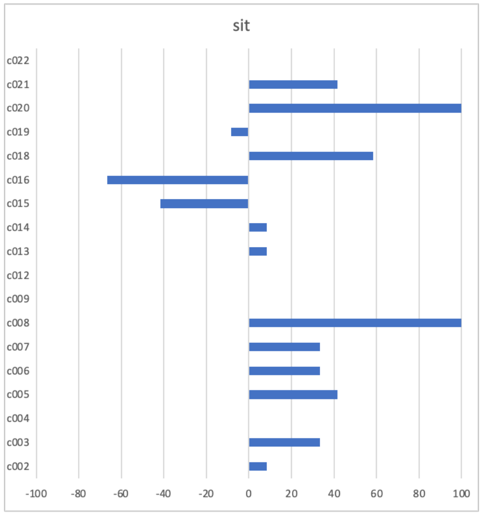

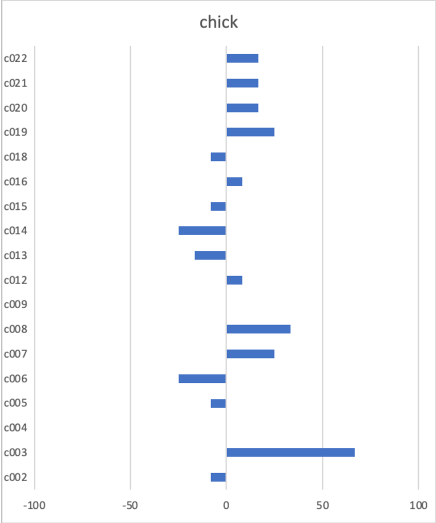

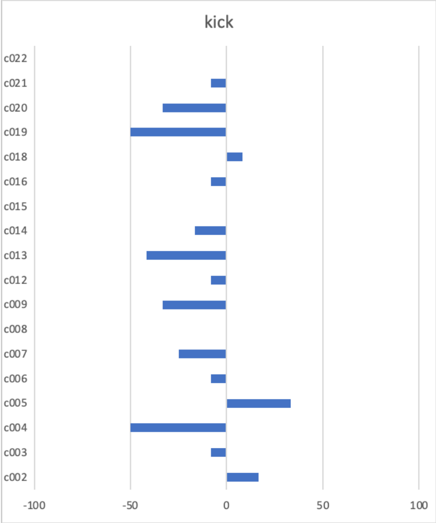

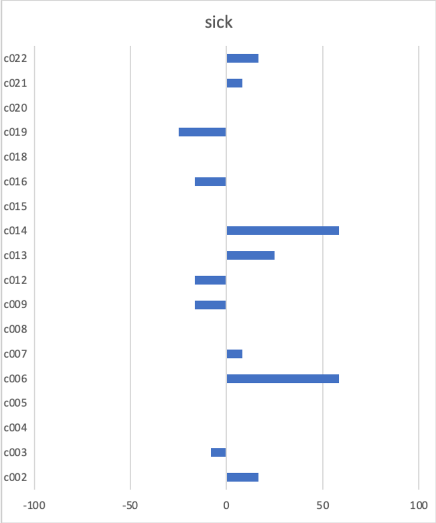

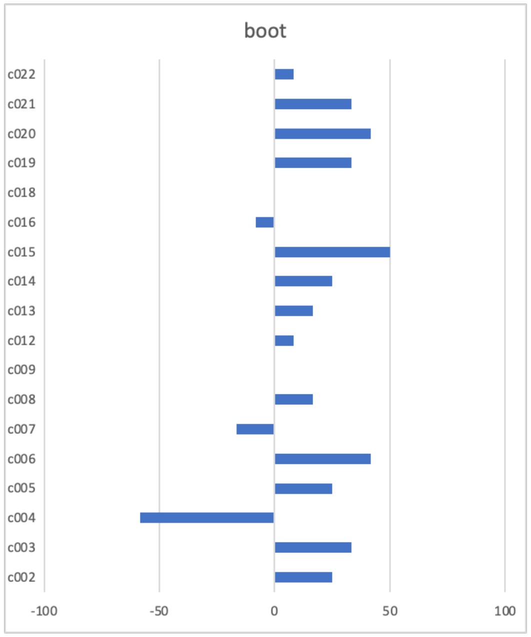
boot
c022
c021
c020
c019
c018
c016
c015
c014
c013
c012
c009
c008
c007
c006
c005
c004
c003
c002
-100
-50
0
50
100

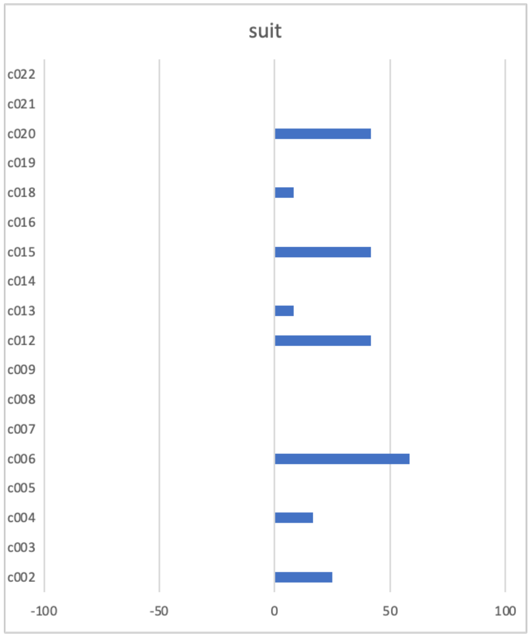
suit
c022
c021
c020
c019
c018
c016
c015
c014
c013
c012
c009
c008
c007
c006
c005
c004
c003
c002
-100
-50
0
50
100

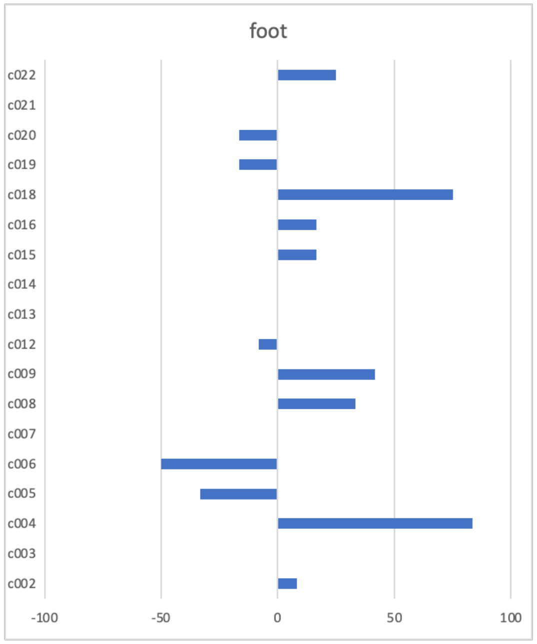
foot
c022
c021
c020
c019
c018
c016
c015
c014
c013
c012
c009
c008
c007
c006
c005
c004
c003
c002
-100
-50
0
50
100

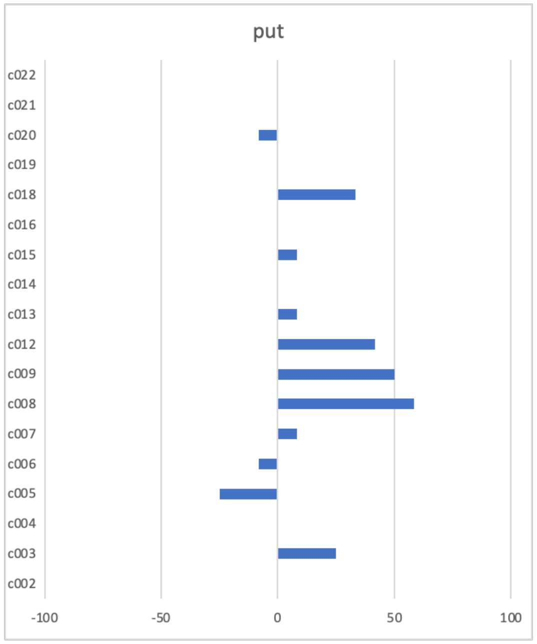
put
c022
c021
c020
c019
c018
c016
c015
c014
c013
c012
c009
c008
c007
c006
c005
c004
c003
c002
-100
-50
0
50
100

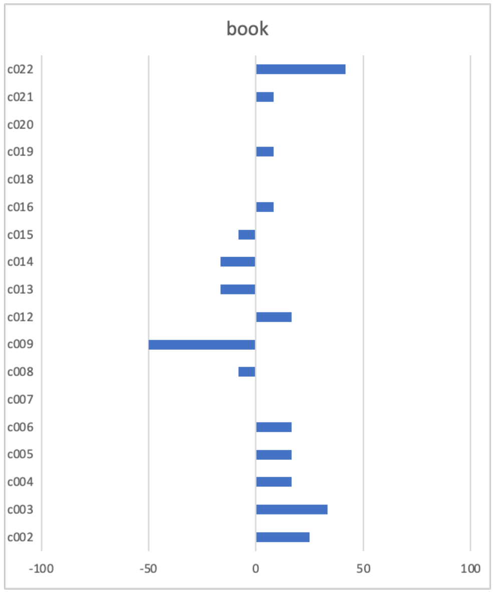
book
c022
c021
c020
c019
c018
c016
c015
c014
c013
c012
c009
c008
c007
c006
c005
c004
c003
c002
-100
-50
0
50
100

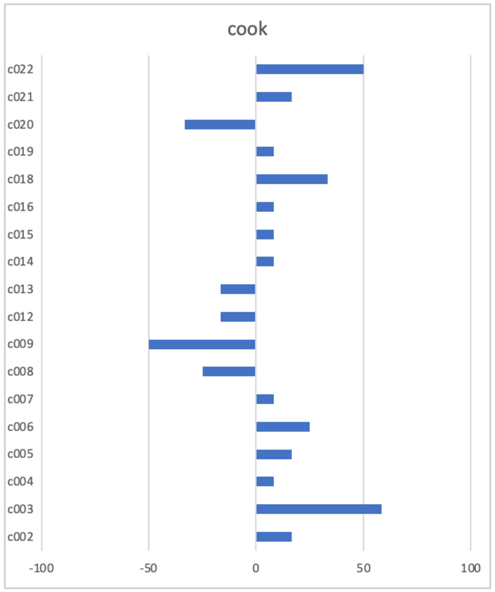
cook
c022
c021
c020
c019
c018
c016
c015
c014
c013
c012
c009
c008
c007
c006
c005
c004
c003
c002
-100
-50
0
50
100

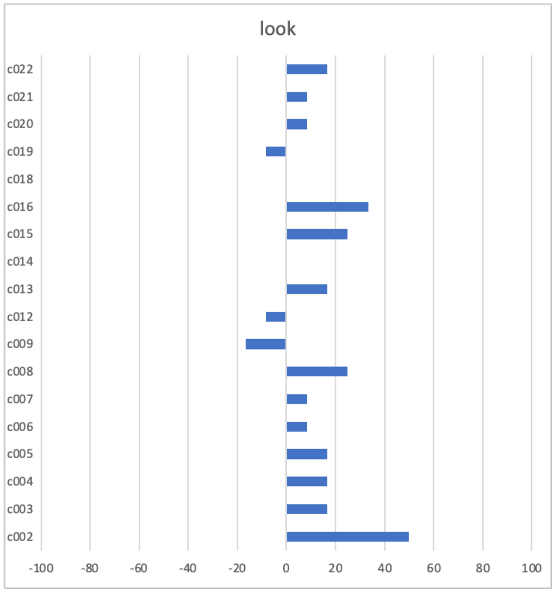
look
c022
c021
c020
c019
c018
c016
c015
c014
c013
c012
c009
c008
c007
c006
c005
c004
c003
c002
-100
-80
-60
-40
-20
0
20
40
60
80
100

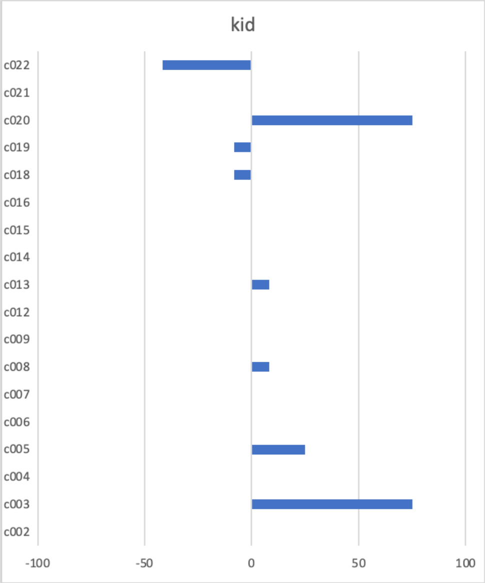
kid
c022
c021
c020
c019
c018
c016
c015
c014
c013
c012
c009
c008
c007
c006
c005
c004
c003
c002
-100
-50
0
50
100

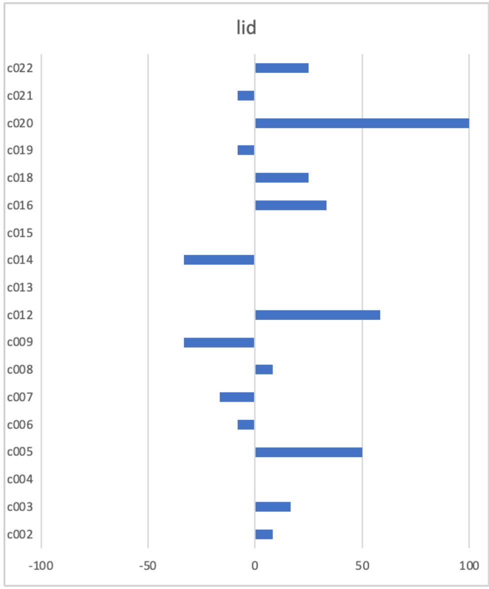
lid
c022
c021
c020
c019
c018
c016
c015
c014
c013
c012
c009
c008
c007
c006
c005
c004
c003
c002
-100
-50
0
50
100

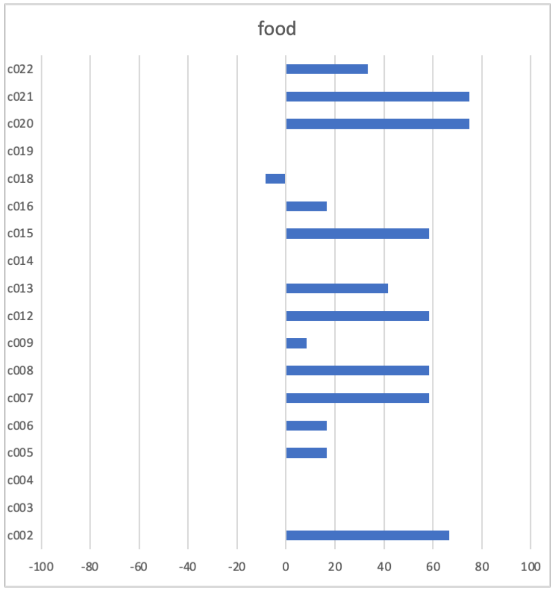
food
c022
c021
c020
c019
c018
c016
c015
c014
c013
c012
c009
c008
c007
c006
c005
c004
c003
c002
-100
-80
-60
-40
-20
0
20
40
60
80
100

Scatterplot showing the relationship between intelligibility scores on the two tasks

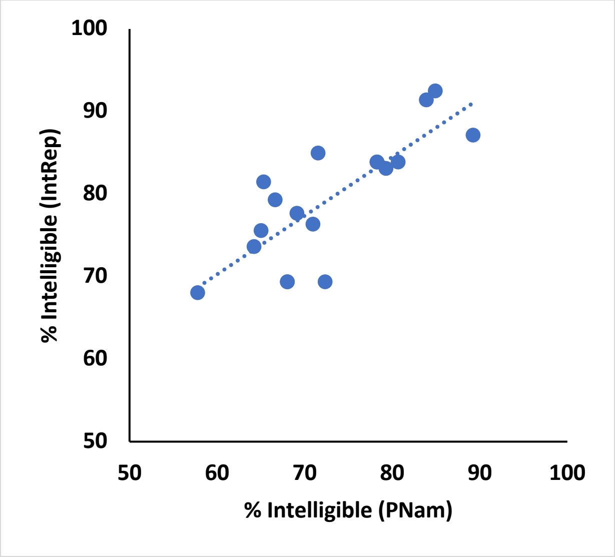

Scatterplot showing the relationship between intelligibility in PNam and the difference in performance on the two tasks

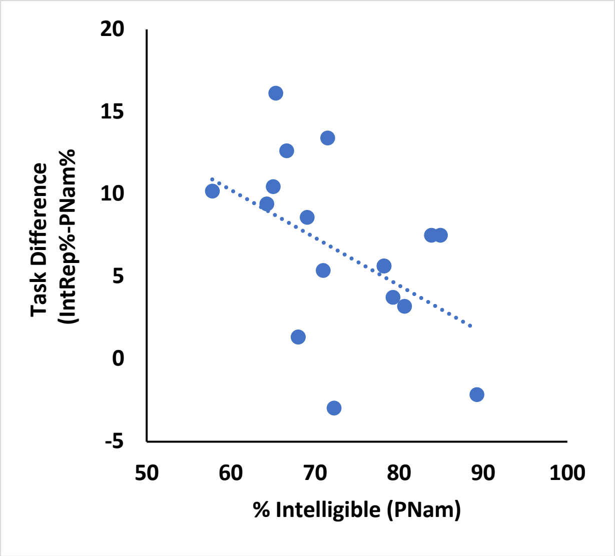

Supplement: Supplementary file 1 [file Data_Sheet_1.PDF]
